# Obesity and breast density enhance immune exclusion in the primary tumor microenvironment and promote breast cancer metastasis

**DOI:** 10.1038/s41388-026-03718-8

**Published:** 2026-02-28

**Authors:** Abbey E. Williams, Erica J. Hoffmann, David R. Inman, Metti K. Gari, Changyan Zhou, Brian M. Burkel, Nour Haidar, Yueran Pan, Megan Halambeck, Brittney N. Moore, Kari B. Wisinski, Stephanie M. McGregor, Sheena C. Kerr, Lisa M. Arendt, Suzanne M. Ponik

**Affiliations:** 1 Department of Comparative Biosciences, School of Veterinary Medicine, University of Wisconsin-Madison, Madison, WI USA; 2 Department of Cell and Regenerative Biology, School of Medicine and Public Health, University of Wisconsin-Madison, Madison, WI USA; 3 Department of Medicine, School of Medicine and Public Health, University of Wisconsin-Madison, Madison, WI USA; 4 University of Wisconsin-Carbone Cancer Center, Madison, WI USA; 5 Department of Pathology and Laboratory Medicine, University of Wisconsin-Madison, Madison, WI USA

**Keywords:** Breast cancer, Cancer microenvironment, Tumour immunology

## Abstract

Recent epidemiological studies suggest that breast density and obesity together increase breast cancer risk. Although these risk factors have been explored individually, little is known about how they combine to alter the tumor immune microenvironment (TIME) and promote disease progression. To address this gap, we developed a murine model of both risk factors. Spatial analysis of the TIME revealed macrophages and T-cells predominantly localized in the stroma of both risk factor groups, indicating an immune exclusion phenotype. Mice with dual risk factors had significantly increased lung metastasis. To establish the human relevance of this model, we interrogated the TIME in biopsies from 158 patients with invasive ductal carcinoma and 10 years of follow-up data. We found that patients with both risk factors had the highest incidence of metastasis (45%). Furthermore, spatial immune profiling revealed exacerbated stromal localization of macrophages and T-cells in the dual risk factor group that progressed to metastasis. Overall, we uncovered an immune exclusion phenotype in metastatic breast cancer patients with obesity and breast density, and we present a relevant murine model that parallels human disease. The murine model will enable future investigation into therapies that intercept the mechanisms by which dual risk factors modulate the TIME.

## Introduction

Breast density and obesity are major individual risk factors for breast cancer [1–3]. These two risk factors are inversely related and thought to confound each other’s effects [3, 4]. However, some women do exhibit both risk factors. In a recent large epidemiological study, obesity and breast density together increased breast cancer risk in Korean women [5]. Further, increased breast density was associated with a higher risk for estrogen receptor-negative breast tumors in women with obesity [6]. These studies suggest that the combined risk factors of breast density and obesity may further enhance breast cancer risk. However, once cancer occurs, the impact of both risk factors on breast cancer progression has not been fully explored.

It is well established that breast density and obesity are independently associated with chronic inflammation in the breast tissue microenvironment. Chronic inflammation has been implicated in the development of multiple cancer types [7], and may enhance breast cancer progression. In humans, dense breast tissue is more inflammatory [8], having elevated levels of extracellular IL-6, IL-8, and CCL5, as well as increased CD45+ immune cells and CD68+ macrophages [9, 10]. Obesity also increases inflammation in breast tissue through the chronic recruitment of macrophages [11]. Macrophages surround necrotic adipocytes and form crown-like structures (CLS), which are a hallmark of obesity, and secrete inflammatory cytokines, including IL-6 [12]. While both breast density and obesity enhance inflammation within normal breast tissue, how these risk factors impact the TIME and impinge on breast cancer metastasis has yet to be identified.

To elucidate alterations in the TIME due to obesity and breast density, we developed a murine model of diet-induced obesity in MMTV-PyMT mice on a background of wild-type (WT) collagen or collagen-dense (HD) mammary tissue. We identified immune exclusion in the primary tumor microenvironment, including a significant reduction in F4/80+ macrophages and CD8 + T-cells in the tumor nest of individual and dual risk factor mice. Strikingly, the dual risk factor group had significantly increased lung metastasis. Using a tissue microarray (TMA) of invasive ductal carcinoma (IDC), we show that patients with both risk factors at the time of breast cancer diagnosis had a higher incidence of metastasis compared to patients with obesity or breast density as a single risk factor or with neither risk factor. Patients with both risk factors who developed metastases showed significantly fewer CD68+ macrophages and CD8 + T-cells in the tumor nest than in the surrounding stroma, suggesting immune exclusion may drive disease progression. Thus, our murine model parallels human disease and presents an opportunity to identify interventions to reduce metastasis for patients with dual risk factors.

## Materials and methods

### Human breast cancer tissue microarray

The IDC TMA was constructed with 176 de-identified patient tissues and consented clinical patient information (minimum of 10 years of follow-up) by Dr. Kari Wisinski and the UW TRIP laboratory (IRB approval 2010-0405). Detailed clinical information of the TMA is outlined in Tables S1-2, and Figs. 3–4.

### Spatial immune analysis

A subset of 32 metastatic and 15 non-metastatic randomly selected TMA cases were profiled using Bruker Spatial Biology GeoMx digital spatial protein profiling. N-counter data were evaluated for signal detection above a threshold of control antibodies (Histone H3 and Ribosomal protein S6), resulting in 43 cases for analysis (Fig. 4).

### Mouse models

**Fig. 1:**
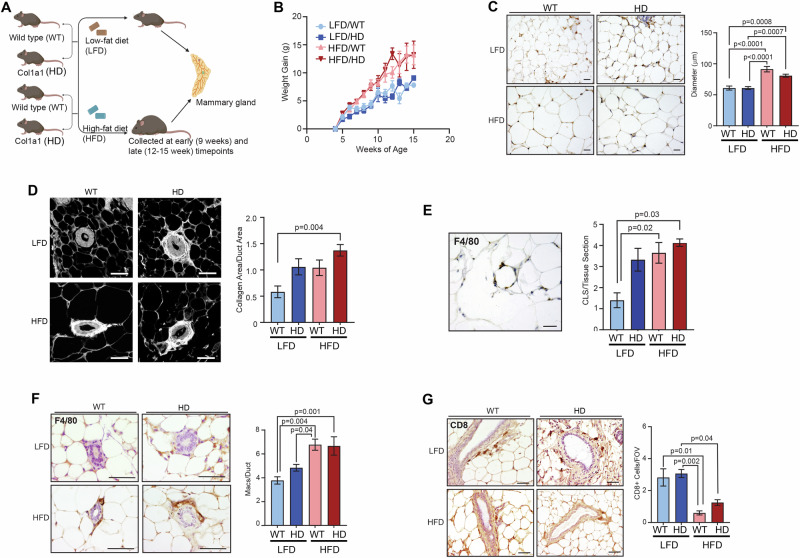
Characterization of mammary glands from non-tumor-bearing obese and collagen-dense mice. **A** Wild type (WT) mice were crossed with *Co1α1* (HD) mice. Starting at 3 weeks of age mice were fed a low-fat diet (LFD), consisting of 9% fat, 44.9% carbohydrates, and 19.0% protein (3.3 kcal/g) (Envigo TD.2019) or a high-fat diet (HFD) consisting of 34.9% fat, 25.9% carbohydrates, and 23.1% protein (5.1 kcal/g)(TestDiet 58Y1). The mice were allowed unrestricted access to their respective diets. Mammary glands were collected from each cohort (LFD/WT, LFD/HD, HFD/WT, or HFD/HD) at the early (9 weeks) or late (12-15 weeks) timepoints. Mice were further divided for analysis of multiple endpoints, with a minimum of N = 4 mice per cohort to to detect a minimum difference of 1.5 standard deviation (SD), with 80% power and a two-sided type I error rate of 5%. **B** Comparison of the average weight gain over time in all four experimental groups (N = 13-14 mice/group). **C** Representative images of mammary adipose tissue for all risk factor groups. Quantification of adipocyte size revealed a significant increase in the diameter of adipocytes in the HFD vs LFD groups at early timepoints (similar results were identified at late timepoints. Data not shown). (N = 4-6 mice/group). **D** Representative images of picrosirius red (collagen) staining of mammary ducts in the fat pads of each risk factor group. Quantification of collagen surrounding mammary ducts shows a significant increase in collagen in the dual risk factor group compared to WT/LFD mammary glands. Data is presented for early timepoints as collagen area per duct area from a minimum of 5 fields of view per tissue section. (N = 4-6 mice/group) **E** Crown-like structures (CLS) of F4/80+ macrophages surrounding adipocytes were quantified per tissue section in mammary glands from late timepoints (N = 4-6 mice/group). CLS increases in LFD/HD mice but are significantly elevated in the HFD/HD group at both late timepoints. **F** Representative images of F4/80+ macrophages surrounding mammary ducts from each risk factor group at late timepoints. Quantification of F4/80+ macrophages around mammary ducts is shown as an average number of macrophages per duct (N = 6-8 mice/group). **G** Representative images of CD8 + T-cells in the mammary glands of each risk factor group. Quantification of CD8 + T cells reveals a significant decrease in CD8+ cell number in the HFD vs LFD groups at the late timepoints (N = 4-8 mice/group). Data is represented as an average +/- standard error of the mean (s.e.m.) of CD8+ cells per field of view (FOV) in the adipose tissue surrounding mammary ducts. Magnification bars **C:** 100 µm, **D**, **E**, **G**: 50 µm, **F**: 25 µm.

**Fig. 2:**
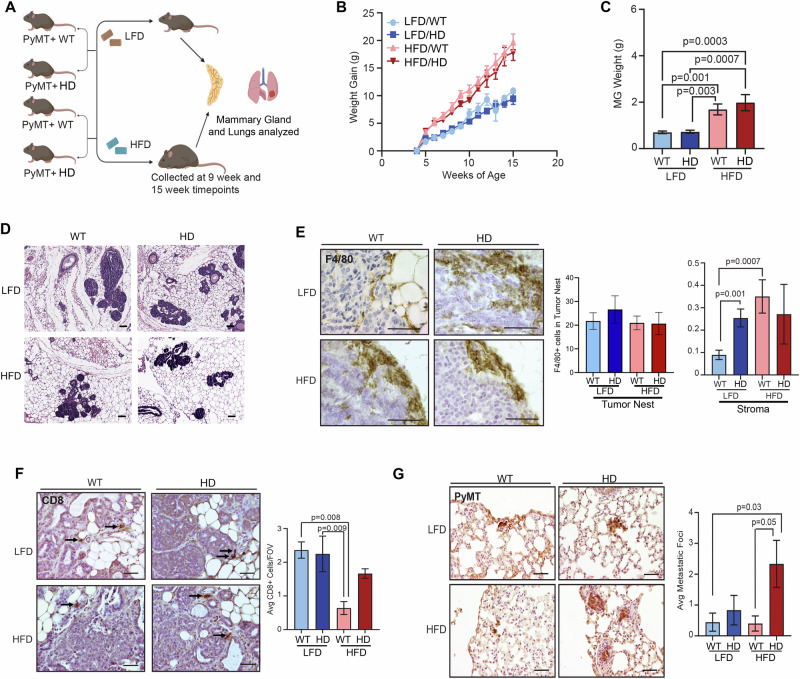
The effect of breast cancer risk factors on tumor initiation and immune exclusion in the MMTV-PyMT mouse model. **A** MMTV-PyMT (PyMT + ) mice were crossed with *Co1α1* (HD) mice. Starting at 3 weeks of age, PyMT+ WT and PyMT+ HD littermates were fed either LFD or HFD. N = 11-20 mice/group. Mice were further divided for analysis of multiple endpoints, with a minimum of N = 5 PyMT+ mice per cohort. Mammary glands and lungs were collected from each risk factor group at either 9 or 15 weeks. **B** Comparison of the average weight gain over time for each group (N = 11-20 mice/group). HFD-fed groups gained more weight than LFD-fed groups by 6 weeks. **C** Mammary gland weight of mice at 9-week timepoint is significantly more in the HFD-fed vs LFD-fed groups (N = 6-10 mice/group). **D** Representative H&E images of the mammary glands at early timepoints show qualitative similarities in hyperplasia and multifocal intraepithelial neoplasia (MIN) in each of the risk factor groups. **E** Representative images of F4/80+ macrophage localization to the tumor boundary at late timepoints (N = 5-10 mice/group). Quantification reveals low abundance of F4/80+ cells within the tumor nest, which is not altered by HFD or HD. However, F4/80+ cells localized at the tumor-stromal boundary are significantly elevated in the LFD/HD and HFD/WT groups compared to LFD/WT tumors. **F** Representative images of CD8 + T cells in adenocarcinomas from mice at 15-week timepoint. All CD8 + T-cells identified localize in stroma regions. The average number of CD8+ cells per field of view (FOV) per gland (N = 5-6 mice/group) was significantly decreased in the HFD/WT group compared to LFD-WT and LFD-HD fed mice. **G** Immunohistochemistry images of PyMT+ cells in the lung tissue of mice from each risk factor cohort. Quantification of the average number of metastatic foci was significantly increased in lung metastasis identified in the dual risk factor group at the late timepoint. Magnification bars **D**, **F**, **G:** 50 µm, **E** 25 µm.

Mice were bred and maintained at the University of Wisconsin-Madison under the approval of the University of Wisconsin Animal Care and Use Committee (protocol: M005840-R02-A01). Transgenic mice expressing the MMTV-PyMT in the FVB background (from The Jackson Laboratory, Bar Harbor, ME, USA) were crossed to female mice heterozygous for the *Col1α1* mutation in the C57BL/6/129 background (a kind gift from Dr. Rudolf Jaenisch). The resulting mice were evenly divided into low- or high-fat diet groups for further analysis (Figs. 1A and 2A).

### Immunohistochemistry

Paraffin-embedded sections of mammary glands from PyMT- and PyMT+ mice were stained by H&E or immunohistochemistry (antibodies list - Table S3). The average cell type or structure was quantified in five images per section. Picrosirius red staining (PSR) was completed as described [13]. All images were taken with a Nikon Eclipse E600 Microscope and QICAM Fast 1394 camera (Teledyne Photometrics, Tuscon, AZ, USA) and analyzed using ImageJ.

### Statistical analysis

For TMA data, BMI and obesity scores for all patient samples were analyzed by linear regression. GeoMX profiling of tumor nest vs tumor stroma and differences in the murine model were determined by nested two-way analysis of variance (ANOVA) Tukey’s multiple comparisons post-test. All results are reported as the mean ± standard error of the mean. unless otherwise noted. A p-value of ≤0.05 denotes a significant value. All statistical analyses were non-blinded and performed with GraphPad Prism 9.4.1.

### Ethics approval and consent to participate

All methods were conducted in accordance with the University of Wisconsin-Madison Biosafety guidelines and regulations (ID: B00000304). Mice were bred and maintained at the University of Wisconsin-Madison under the approval of the University of Wisconsin Animal Care and Use Committee (protocol: M005840-R02-A01). The IDC TMA was constructed with 176 de-identified patient tissues and consented clinical patient information (minimum of 10 years of follow-up) by Dr. Kari Wisinski and the UW TRIP laboratory (IRB approval 2010-0405). There are no identifiable images in the manuscript.

## Results and discussion

### Dual risk factors enhance macrophage recruitment and diminish CD8 + T cells in the mammary glands of non-tumor-bearing mice

To investigate the immune microenvironment of the dual risk factor mouse model, we examined the mammary glands of non-tumor-bearing mice (PyMT-). As described in our prior studies [14–17], we utilize Col1α1 mutant mice (HD mice) to model mammographic density. WT and HD littermates were randomized to receive either LFD or HFD starting at 3-weeks of age (Fig. 1A). As expected, both WT and HD mice fed HFD gained more weight than mice fed LFD (Fig. 1B). At the early and late timepoints, the HFD-fed groups had significantly higher body mass (Fig. S1A, B) and mammary gland weight (Fig. S1C, D) than their LFD-fed counterparts. Consistent with increased mammary gland weights, at early timepoints the mammary glands of HFD-fed WT and HD mice had significantly larger adipocytes (Fig. 1C).

Although mammographic density and obesity have an inverse relationship [3, 4], we recently observed that obesity enhances collagen deposition in the mammary gland [18]. To understand how the dual risk factor model contributes to collagen deposition, we quantified collagen surrounding mammary ducts (Fig. 1D). The dual risk factor mice had significantly greater collagen deposition around ducts compared to LFD-fed WT mice (Fig. 1D). Notably, there was a non-significant trend towards increased collagen surrounding ducts in the HD group compared to LD group for both LFD and HFD cohorts (Fig. 1D). Overall, the HFD-fed WT and HD groups showed increased mammary gland weight, adiposity, and collagen surrounding mammary ducts.

It is established that mammary tissues from healthy women with either high mammographic density or obesity have increased inflammation [8, 10–12, 19]. To determine the impact of dual risk factors on inflammation in our mouse model, we identified F4/80 + CLS within the mammary glands (Fig. 1E). While LFD-fed HD mammary glands trend toward increased CLS compared to LFD-fed LD mice, due to heterogeneity, the increase was only significant in HFD-fed WT and HD mice compared to WT LFD-fed mice (Fig. 1E). In addition to CLS, we also examined macrophages directly in contact with ductal epithelial cells (Fig. 1F). Again, there is a trend toward increased periductal F4/80+ macrophages in LFD-fed HD vs WT mice. However, only the HFD-fed groups (WT and HD) had a significant increase in periductal macrophages compared to LFD-fed mice (Fig. 1F).

Increased macrophages and collagen are thought to have suppressive effects on the recruitment of CD8 + T cells [20–22]. Based on the increase in collagen and macrophages in CLS and in periductal regions of the dual risk factor group, we sought to examine CD8 + T cell recruitment within mammary glands (Fig. 1G). Overall, we detected very few CD8+ cells irrespective of the risk factor group. Collagen density did not alter the number of CD8 + T cells, however, there was a striking reduction in CD8 + T cell recruitment in the mammary glands in response to HFD (Fig. 1G). To summarize, compared to LFD-fed WT mice, the HFD-fed mice had increased CLS and macrophages surrounding ducts in the mammary glands of non-tumor-bearing mice. The increase in macrophages does not directly correlate with CD8 + T-cell recruitment in all conditions, but rather obesity is a key driver in the suppression of CD8 + T-cell recruitment to the mammary gland. This may suggest that there are functional differences in macrophage subtypes found in tumors associated with breast density and obesity, and further work is necessary to explore the spatial relationship between these functional subtypes and T-cell response.

### Obesity and mammary density promote immune exclusion in the MMTV-PyMT TIME and enhance lung metastasis

To model how the risk factors of obesity and mammographic density impact mammary tumors, WT and HD mice were crossed with MMTV-PyMT+ (PyMT + ) mice [23]. PyMT+ WT and HD littermates were randomized to receive either LFD or HFD starting at 3 weeks of age (Fig. 2A). By 6-weeks the HFD-fed cohorts (PyMT+ WT and HD) gained more weight than LFD-fed mice (Fig. 2B). At the 9-week timepoint, body weight (Fig. S2A) and mammary gland weight (Fig. 2C) of PyMT+ HFD-fed cohorts were significantly higher than LFD-fed cohorts. The body weights of the PyMT+ mice at both timepoints were similar to the body weights of PyMT- mice at corresponding timepoints (Fig. S2B), demonstrating that PyMT transgene expression did not alter weight gain in these cohorts of mice.

Early in tumor progression, PyMT+ mice are known to develop multifocal intraepithelial neoplastic lesions (MIN) within their mammary glands [24]. Diet-induced obesity has been shown to increase tumor size in MMTV-PyMT mice, suggesting that MIN progresses to tumors at a higher rate in HFD-fed mice [25]. Similarly, our group and others have reported enhanced tumor growth and metastasis in the Col1α1 mice [15, 26, 27]. However, tumor initiation and early progression have not been assessed in HD mice nor in dual risk factor mice. To address this gap, we assessed hyperplasia, MIN, and early-stage in each cohort. H&E staining revealed heterogeneous regions of hyperplasia, MIN, and invasive adenocarcinoma within the mammary glands of each cohort (Fig. 2D). No significant differences in percentage of hyperplasia or MIN per gland were identified between cohorts (Fig. S2C). Overall, neither individual nor the combination of risk factors affected the initiation or development of early-stage neoplastic lesions in the PyMT+ model.

Based on the differential immune infiltration in dual risk factor cohorts of non-tumor-bearing mice (Fig. 1E-G), we hypothesized that our model of obesity and collagen density would induce similar changes in immune infiltration in primary tumors. Our group has previously observed that these individual risk factors impact the TIME, such that HFD-fed mice had increased macrophages surrounding areas of hyperplasia and tumors in a Trp53^-/-^ model of mammary tumorigenesis [28]. Similarly, we have identified an increase in macrophages in MMTV-PyMT tumors that arise in HD vs. WT mammary glands [27]. Here, we detect no difference in F4/80+ cells surrounding regions of MIN at early timepoints with either risk factor (Fig. S2D). At late tumor timepoints, we quantified relatively low numbers of F4/80+ macrophages within the tumor nest in all cohorts (Fig. 2E and S2E). In contrast, we observed robust localization of macrophages at the tumor-stromal boundary (Fig. 2E). Specifically, macrophages in the stroma of LFD-fed HD mice were significantly elevated compared to the LFD-fed WT controls, and HFD-fed WT mice also had an increase in F4/80+ macrophages in the stroma compared to LFD-fed WT mice (Fig. 2E). Macrophages surrounding tumors in HFD-fed HD mice are also elevated, but due to the heterogeneity in this cohort, the increase did not achieve significance (Fig. 2E). Next, we assessed the abundance and spatial localization of CD8 + T-cells. We identified very low numbers of CD8+ cells in every cohort. Similar to published findings [29, 30], HFD further reduced CD8+ cells. The reduction was significant in the WT, HFD cohort compared to both LFD-fed groups (WT and HD) (Fig. 2F). The CD8 + T-cells that were detected were localized in stromal regions, demonstrating an immune exclusion phenotype (Fig. 2F).

Finally, we sought to determine how changes in the immune microenvironment relate to metastatic progression. To this end, metastatic lung lesions were identified using an antibody against PyMT. The number of metastatic foci was significantly increased in the dual risk factor group compared to WT mice in either the LFD- or HFD-fed groups (Fig. 2G). Thus, we present a pre-clinical model of breast cancer risk factors that demonstrate obesity and collagen density regulate the TIME, and the combination of these risk factors promotes breast cancer metastasis.

### Characteristics of invasive ductal carcinoma patients with obesity and breast density

**Fig. 3:**
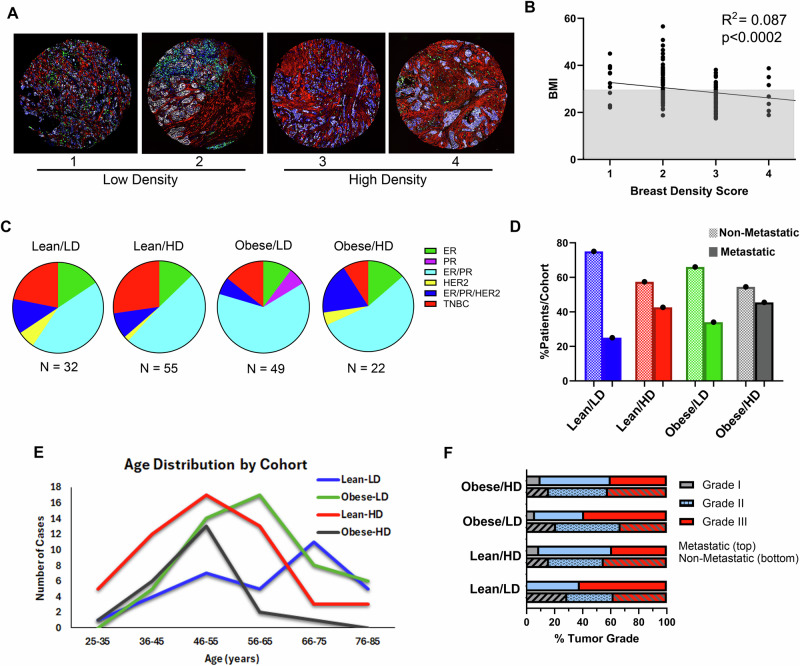
Assessment of obesity and breast density in a retrospective TMA with associated clinical database of IDC patient cases. **A** Representative immunofluorescent images of IDC patient biopsy tissues with breast density scores ranging from 1 – 4 (1 = fatty, 2 = scattered fibroglandular, 3 = heterogeneously dense, and 4 = dense or extremely dense). Collagen = red, Pan-cytokeratin = white, CD45 = green, and DAPI = blue. Two 1.0 mm diameter biopsy punches per case. **B** 158 cases were scored for BMI ((BMI = weight (lbs) / height (in^2^) x 703, with BMI ≥30 = obese, <30 = lean) and breast density (score 1-2 = low density, score 3-4 = high-density) and assessed by Spearman’s correlation. Similar to prior reports, BMI and breast density are inversely correlated. **C** Pie charts depict the distribution of hormone receptor status for each risk factor cohort (Lean/LD, Lean/HD, Obese/LD, and Obese/HD). N = number of cases in each cohort. **D** Percentage of non-metastatic (shaded bars) and metastatic (solid bars) TMA cases within each risk factor cohort. **E** The distribution of patient age at diagnosis for each cohort. The dual risk factor cohort has the youngest age distribution compared to cohorts with single risk factors (Lean/HD and Obese/LD) or no risk factors (Lean/LD). **F** Histologic assessment of tumor grade at diagnosis extracted from the clinical data reveals no marked difference in the distribution of tumor grade between risk factor cohorts.

To validate the human relevance of our murine model, we assessed a retrospective database of patient cases with associated clinical data (Tables S1 and S2). The distribution of breast cancer subtypes in the TMA (67.7% Luminal A (ER + /PR + /HER2-), 10.1% Luminal B (ER/PR/HER2 + ), 2.5% HER2 + , and 19.6% TNBC) is representative of subtypes observed clinically [31]. First, we categorized each case by BMI and mammographic density score. Density score was assigned a numerical value of 1 – 4 (Fig. 3 and Tables S1). 8 benign cases and 10 cases with unknown density score were removed from the study. Collagen accumulation and epithelial changes were assessed by immunofluorescence; representative images of each density score are shown in Fig. 3A. Similar to previous reports [3, 4], breast density and BMI are inversely related (Fig. 3B). Despite the inverse correlation, 22 patients were identified as having both risk factors (obesity, BMI ≥ 30; HD, score = 3-4), 55 with HD alone, 49 with obesity alone, and 32 patients had neither risk factor. The distribution of breast cancer subtypes in each of the 4 cohorts, Lean/LD, Lean/HD, Obese/LD, and Obese/HD, shows predominantly hormone receptor-positive cases with a decreased number of TNBC cases in the obese cohorts (Fig. 3C and Table S2). This aligns with recent studies showing increased aggressive breast cancer subtypes (HER2+ and TNBC) in patients with high density and high BMI [32]. Without considering breast cancer subtype, we identified that the percentage of metastatic patients (solid bars) (Fig. 3D) was highest in the dual risk factor group (45.5%) compared to obesity (34%) or HD (42.6%) alone. Analysis of the hormone-positive cases revealed 47% of the obese/HD cohort progressed to metastasis compared to either single risk factor (obesity = 31% and high density = 41%) (Fig. S3). Interestingly, the age distribution for the dual risk factor cohort trended toward a younger peak age at diagnosis, with few patients over the 55-65 age range compared to the other cohorts (Fig. 3E). This is consistent with the known decrease in mammographic density with age [33]. Tumor grade at diagnosis was similar between metastatic and non-metastatic cases across all four cohorts (Fig. 3F and Table S1). These data support an increase in metastatic breast cancer in patients with both HD and obesity.

### Immune exclusion in the primary tumor microenvironment of patients with obesity and breast density is associated with metastasis

**Fig. 4:**
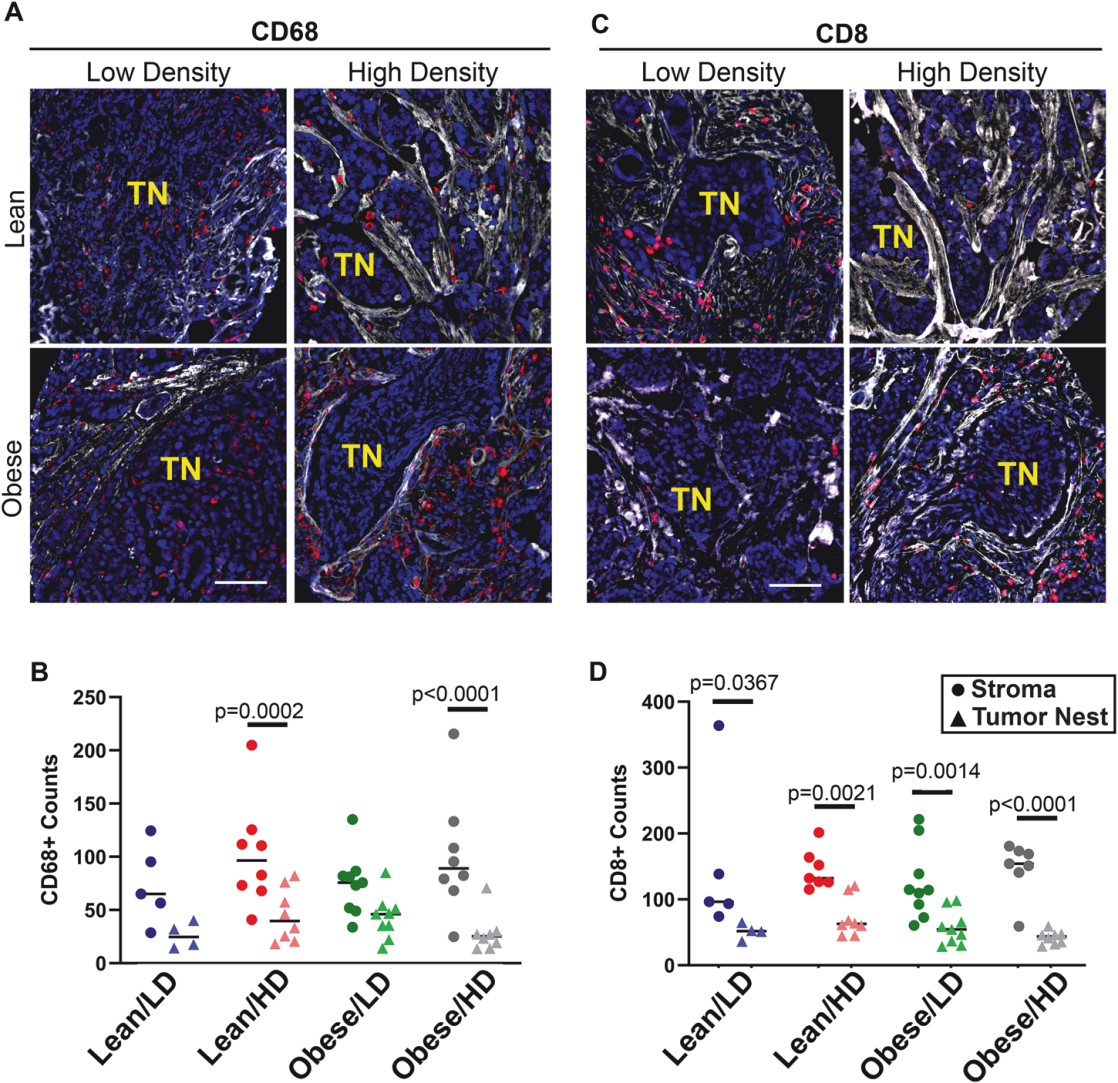
Obesity and high breast density promote immune exclusion in primary IDC. **A**, **B** Representative immunofluorescence images of the spatial localization of immune cells in the tumor nest vs tumor stroma (collagen-rich regions). **A** CD68+ macrophages and **(B)** CD8 + T-cells in biopsy tissue from IDC cases that present with each risk factor (Lean/LD, Lean/HD, Obese/LD, and Obese/HD). CD68+ (A) or CD8+ (B) cells = red, collagen = white, and DAPI = blue. TN (yellow) identifies the tumor nest. Magnification bar: 50 µm. **C**, **D** Bruker Spatial Biology GeoMx profiling of 8 lean/LD, 13 lean/HD, 13 Obese/HD, and 9 Obese/HD cases provided quantification of (**C**) CD68+ or (**D**) CD8+ counts localized within the collagen stromal matrix or in the tumor nest (TN). The TMA was stained with fluorescent antibodies against pan-cytokeratin, CD45, collagen, and the nuclear stain DAPI, followed by barcoded antibodies (GeoMX Immune Profile and Immune Cell Typing panels). Regions of interest (ROIs) for GeoMX segmentation were visually selected to include approximately 50% tumor nest (PanCK) and 50% stroma (collagen). Each data point represents the average of two regions of interest (ROI) taken from two separate biopsies per patient. p-value analyzed by nested two-way Anova, comparison of GeoMX counts in the stroma vs tumor nest.

Based on the importance of the breast TIME to metastatic progression [34, 35], we defined how obesity and breast density independently and cooperatively impinge on the tumor immune response. Using spatial proteomic analysis of immune populations analyzed with Bruker Spatial Profiling GeoMX, we identified an immune exclusion phenotype in metastatic patients with HD at the time of diagnosis (Fig. 4). Specifically, we visualized CD68+ macrophages localized to the collagen-rich stroma (Fig. 4A). GeoMX analysis revealed that the CD68+ macrophages were largely absent from the tumor nest, only in metastatic patient samples (Figs. 4B and S4A). The alternatively activated or anti-inflammatory (M2) macrophage marker CD163 trended toward immune exclusion, but the localization of this marker did not achieve statistical significance in either the metastatic or non-metastatic cohort (Fig. S4B-C). Similar to CD68+ cells, CD8 + T-cells were excluded from the tumor nest but detected in the collagen-rich stroma of metastatic patient samples (Fig. 4C, D), while CD8+ cells in non-metastatic samples had no distinct spatial localizations (Fig. S4D). GeoMX analysis of immune cell exclusion from the tumor nest was even more evident in cases where both risk factors were present at diagnosis (Fig. 4B, D). Specifically, the average fold difference of CD68+ cells localized in the tumor stroma vs tumor nest was 3.7-fold in the dual risk group, compared to 3.4-, 2.7-, and 1.9-fold differences in lean/LD, lean/HD, and obese/LD, respectively. Overall, the CD8 + T-cell localization in the stroma vs tumor nest was 3.9-fold in dual-risk cases, compared to 2.0-, 2.5-, and 3.1-fold differences in lean/LD, lean/HD, and obese/LD, respectively.

Due to the random selection of cases for GeoMX profiling, only one non-metastatic case was selected from the dual risk factor group, and therefore, statistical significance could not be determined. However, within the limited case number, immune exclusion was not observed in non-metastatic cases (Fig. S4A, C, &D). Taken together, these findings suggest that the combination of obesity and breast density drives immune exclusion in primary human lesions and is associated with increased progression to metastasis.

## Conclusions

To better understand the influence of breast density and obesity on the immune microenvironment and disease progression, we established a murine model of mammary tissue density paired with diet-induced obesity. In this model, these risk factors promote an immune exclusion phenotype in the tumor microenvironment, and the combination of risk factors increases lung metastasis. Similarly, patients with obesity and breast density at diagnosis have an enhanced immune exclusion phenotype, which is also associated with an increase in metastasis. Future studies in our murine model will define the mechanisms by which these risk factors modulate the TIME to promote metastasis to reveal novel treatment strategies for patients with breast density and obesity-associated breast cancer.

## Supplementary information

Supplemental Figure Legends

Supplemental Figure 1

Supplemental Figure 2

Supplemental Figure 3

Supplemental Figure 4

Supplemental Table 1A

Supplemental Table 1B

Supplemental Table 2

Supplemental Table 3
