# Supplementary material for: Obesity and breast density enhance immune exclusion in the primary tumor microenvironment and promote breast cancer metastasis
Source: Oncogene. 2026 Feb 28;45(11):1057–64. doi: 10.1038/s41388-026-03718-8 (PMC12987724; doi:10.1038/s41388-026-03718-8)
Supplement: Supplementary file 1 — Supplemental Figure Legends [file 41388_2026_3718_MOESM1_ESM.docx]

SUPPLEMENTARY FIGURE LEGENDS:

**Supplementary Figure 1. Obesity increased mouse body weight and mammary gland weight in non-tumor-bearing mice. A.** Body weight of mice in grams (g) at the early timepoint of 9 weeks (N = 6 mice/group). **B.** Body weight at the late timepoint of 12-15 weeks (N = 6 mice/group).  **C.** Mammary gland weight in grams (g) at the early timepoint (N = 6 mice/group).  **D.** Mammary gland weight from mice at the late timepoint (N = 6-8 mice/group). HFD increased body weight and mammary gland weight in WT and HD mice compared to LFD-fed mice of either genotype.

**Supplementary Figure 2. Characteristics of breast cancer risk factors in the MMTV-PyMT mouse model.**  **A.** Body weight of PyMT+ mice in grams (g) at 9-week timepoint (n= 5-10 mice/group). **B.** Comparison of final body weights of PyMT- and PyMT+ mice at 9-week timepoint (n= 6-10 mice/group) or at the 15-week timepoint (n= 6-8 mice/group). The MMTV-PyMT transgene did not significantly alter body weight in any of the groups. **C.** Quantification of the percentage of hyperplasia and MIN per mammary gland (MG) for each risk factor cohort revealed no change in tumor initiation. Representative H&E images of a hyperplasia or a MIN. Magnification bar: 50 µm **D.** Representative images of early timepoint mammary glands stained for F4/80 surrounding MIN. No significant differences in the number of F4/80+ cells located near MIN were identified. Magnification bar: 50 µm.  **E.** Representative images of late timepoint tumors stained for F4/80+ cells. F4/80+ cells in the tumor nest are not altered by either breast cancer risk factor. Magnification bar: 25 µm.

**Supplementary Figure 3. Percentage of non-metastatic and metastatic hormone receptor-positive (Luminal A) cases.** Selecting hormone receptor-positive cases (ER+ and/or PR+ cases), the percentage of non-metastatic (shaded bars) and metastatic (solid bars) cases is presented for each risk factor cohort. The dual risk factor group has the highest percentage of cases that progressed to metastasis (47%), which the lean/LD (no risk factor group) has the highest percentage of non-metastatic cases (87%).

**Supplementary Figure 4. Immune exclusion is absent from all risk factor groups in the non-metastatic cases.** Spatial profiles of non-metastatic cases reveal no change in localization of **(A)** CD68+ macrophages, **(B)** CD8+ T-cells, or **(D)** CD163+ macrophages in the stroma vs tumor nest. **C.** Quantification of CD163+ macrophages in metastatic cases shows a non-significant trend toward immune exclusion.
